# Quaternary Climate Oscillations Shape Genetic Diversity and Spatial Structure of *Viola hybanthoides* (Violaceae) Endemic to Danxia Landscape

**DOI:** 10.1002/ece3.74043

**Published:** 2026-07-29

**Authors:** Sufang Chen, Yanshuang Huang, Zhiyi Xie, Jianqiang Guo, Fang Chen, Wenbo Liao, Qiang Fan

**Affiliations:** ^1^ State Key Laboratory of Biocontrol and Guangdong Provincial Key Laboratory of Plant Stress Biology, School of Life Sciences Sun Yat‐Sen University Guangzhou China; ^2^ Guangdong Ecological and Environmental Monitoring Center Guangzhou China; ^3^ Guangdong Danxiashan National Nature Reserve Administration Shaoguan China

**Keywords:** conservation strategy, danxia landscape, population genomics, quaternary climate oscillations, *Viola hybanthoides*

## Abstract

*Viola hybanthoides* is a narrowly endemic species restricted to two isolated danxia landscapes (Pingshi and Mount Danxiashan) in Southeast China, serving as an ideal model for studying endemism in fragmented habitats. Using ddRAD‐seq and shallow genome sequencing, we genotyped 156 individuals from 13 populations of the species to explore its genetic patterns and evolutionary processes. Results showed extremely low within‐population genetic diversity (*H*_*E*_ = 0.058 ± 0.028), probably driven by limited pollen and seed dispersal ranges, recurrent founder events, and persistent genetic drift exacerbated by post‐glacial population contractions. Pronounced genetic differentiation existed between Pingshi and Danxiashan, with Danxiashan populations showing an east–west split by the Jinjiang River. Admixture analysis and plastid phylogeny identified one population of Pingshi as a hybrid population between Danxiashan and Pingshi. Combined with population dynamics and fastsimcoal2 inference, it is inferred that *V. hybanthoides* expanded during glacial periods and contracted to refugia in interglacials, with Danxiashan populations spreading northwestward to Pingshi in glaciations, giving rise to secondary contact and hybrid lineages. Thus, the species faces threats including depleted genetic variation, severe habitat fragmentation, and human‐exacerbated historical bottlenecks. We propose targeted conservation strategies, including prioritized in situ protection of core genetically representative populations, population reinforcement via tissue culture and reintroduction, and long‐term multi‐indicator population dynamic monitoring. This study elucidates how Quaternary climate fluctuations and danxia topographic isolation jointly shape the genomic differentiation of endemic plants and provides population‐genomic references for biodiversity conservation in fragmented edaphic island ecosystems.

## Graphical abstract

We adopted ddRAD‐seq and chloroplast genome sequencing to explore the genetic characteristics of the danxia‐endemic species *Viola hybanthoides*. This species exhibits extremely low within‐population genetic diversity and obvious genetic differentiation, which is mainly driven by habitat fragmentation, restricted gene flow and Quaternary climatic changes. We also identified a hybrid population derived from historical population expansion and put forward targeted conservation strategies, providing new insights into the evolutionary adaptation and conservation of danxia endemic plants.

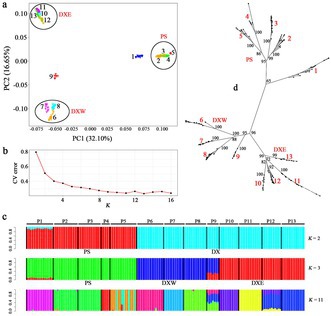

## 1 Introduction

The danxia landscape is a unique geomorphic type endemic to China, characterized by red sandstone cliffs and steep slopes (Peng 2001; Huang et al. 2015). It was designated as the World Natural Heritage “China Danxia” by UNESCO in 2010 (UNESCO 2010). Most danxia outcrops across southern China are scattered and small, with individual patches typically covering less than 10 km^2^ (Yan et al. 2019). In contrast, Mount Danxiashan in Guangdong Province represents an exceptionally large and representative danxia cluster, spanning approximately 292 km^2^ and comprising more than 680 red peaks (Peng 2020). Long‐term weathering, fluvial erosion, and incision have partitioned this terrain into isolated peak clusters, cliffs, and valley systems, forming a fragmented “terrestrial island” landscape with strong topographic isolation (Wu et al. 2008). Danxia landscape habitats are defined by thin, gravel‐rich, infertile soils, intense solar radiation, large diurnal temperature fluctuations, rapid evaporation, and unstable soil moisture conditions (Huang 2009; Peng 2020). These harsh but highly heterogeneous microenvironments create diverse ecological niches and sustain complete vegetation successional sequences (Peng et al. 2018). Accordingly, danxia landscape serves as a valuable natural laboratory for exploring plant community assembly and ecological adaptation (Chen et al. 2008; Hou et al. 2008).

Danxiashan hosts more than 20 newly described plant species, most of which are spatially confined solely to this massif (Fan et al. 2022). Unlike these highly restricted endemics, a small group of specialized taxa, represented by *Primulina* species, exhibit wide edaphic tolerance and successfully colonize multiple danxia and karst landscapes across geographically distinct regions (Wang, Feng, et al. 2017; Feng et al. 2025). Similarly, a few other species such as *Firmiana danxiaensis*, *Diospyros danxiaensis*, and *Viola hybanthoides* are distributed across a handful of geographically isolated danxia patches (Fan et al. 2015; Tong and Xia 2019; Chen et al. 2024). This rare cross‐patch distribution pattern offers an ideal study system for unraveling landscape‐derived dispersal barriers, inter‐population gene flow dynamics, and adaptive differentiation within fragmented terrestrial island ecosystems. Previous genetic and genomic studies on danxia landscape‐associated plants have deepened our knowledge of landscape‐driven plant divergence while exposing critical unresolved issues. For instance, population genetic analyses of the narrow endemic *Primulina danxiaensis* have confirmed fine‐scale spatial genetic differentiation shaped by microtopographic isolation within Danxiashan (Chen et al. 2021). Genomic investigations of *F. danxiaensis*, a species adaptable to both danxia and karst landscapes, have further revealed pronounced interregional genetic differentiation with limited gene flow, as well as subtle east–west genetic structuring across Danxiashan (Chen et al. 2024). At the broader Nanling Mountains scale, phylogenomic studies of the polyphyletic genus *Primulina* have documented historical gene flow between karst and danxia populations (Ke et al. 2022). Despite these valuable findings, fundamental evolutionary and ecological questions for danxia‐endemic plants remain poorly resolved. It remains unclear how endemic taxa disperse among disjunct danxia terrestrial islands, whether secondary contact occurs between geographically isolated populations, and how Quaternary climatic fluctuations have shaped contemporary genetic structure and local adaptation. Addressing these knowledge gaps is essential to clarify how vicariance, dispersal, geographic isolation, and natural selection jointly shape endemism patterns in subtropical fragmented landscapes.

*Viola hybanthoides* W. B. Liao & Q. Fan (Violaceae) is a narrowly distributed danxia endemic species. It was first formally described based on materials collected from Danxiashan (Fan et al. 2015), and our recent field investigations have further discovered its new occurrence at other danxia landscapes in Pingshi, Guangdong, China. The two confirmed distribution localities are separated by around 60 km of continuous mountain terrain, forming a distinct distribution pattern that is geographically isolated yet spatially close. Combined with its strong adaption to dry, infertile cliff habitats and rocky slopes typical of danxia landscapes, this disjunct distribution makes *V. hybanthoides* an excellent study object to explore microevolutionary processes and the formation mechanisms of plant endemism within isolated terrestrial island habitats.

The genus *Viola* possesses relatively clear pollination and seed dispersal characteristics. Most species in this genus produce nectar‐rich chasmogamous flowers mainly pollinated by wild bees or beeflies, while cleistogamous flowers can ensure normal reproductive success under pollinator shortage (Beattie 1974, 1976; Culley 2002). Relevant field studies on multiple *Viola* species have verified that pollinator flight distances are greatly restricted. Valid pollen is mostly transmitted within 3 m, and most outcrossing events occur between individuals less than 30 cm away from each other (Beattie 1976; Peng et al. 2025). Most widespread *Viola* species rely on diplochory to complete seed dispersal. Their seed capsules eject seeds ballistically for 1–2 m, and seed elaiosomes can facilitate secondary dispersal by ants, which further extends the seed spreading distance by 2–3 m (Beattie and Lyons 1975; Ohkawara and Higashi 1994; Sharpe and Ruxton 2025). Moreover, most *Viola* species can achieve clonal reproduction via slender creeping horizontal rhizomes. New shoots and adventitious roots grow from rhizome nodes, forming numerous genetically identical ramets (Bizoux and Mahy 2007; Stace 2010). In sharp contrast, the danxia‐restricted endemic *V. hybanthoides* has evolved differentiated reproductive traits. It develops special *Hybanthus*‐like flowers with functional nectar spurs, which implies potential pollination by native beeflies (Fan et al. 2015). Unlike most congeners, this species does not form seed elaiosomes (personal observations), so its seeds are only spread through ballistic ejection without ant‐mediated secondary dispersal. It develops erect and robust rhizomes that mainly function for nutrient storage (Fan et al. 2015); these structures rarely develop lateral branches for clonal growth, which results in its extremely weak clonal expansion capacity (Klimeš et al. 1997).

A series of population genetic studies using AFLP, ISSR, SSR, ITS, single‐copy orthologs and RAD‐seq markers have summarized universal genetic characteristics of the genus *Viola*. Generally, this genus maintains moderate to high genetic diversity at the species level, but presents obviously lower genetic diversity within populations. Such a pattern is primarily caused by mixed mating systems dominated by self‐pollination and vegetative propagation (Ballard et al. 1998; Hirai et al. 2012; Kuta et al. 2014; Żabicka et al. 2020, 2025). Narrowly endemic *Viola* species with limited dispersal ability and dominant selfing usually show high levels of genetic differentiation among different populations (Auge et al. 2001; Eckstein and Otte 2005). Furthermore, frequent intraspecific hybridization and genetic introgression commonly occur in *Viola*, with barely any genetic barriers separating most species (Marcussen et al. 2022). These findings confirm that population genetic differentiation within this genus is mainly shaped by geographical isolation rather than intrinsic reproductive isolation.

In this study, we adopt a population genomic approach based on double‐digest restriction site‐associated DNA sequencing (ddRAD‐seq) and shallow whole‐genome resequencing (Peterson et al. 2012). We performed genotyping on 156 individuals originating from 13 populations distributed across Danxiashan and Pingshi, and further assembled complete chloroplast genomes for representative samples. By integrating nuclear and plastid genomic data, we address three core research questions: (1) Given the generally limited dispersal ability of *Viola* species, how did ancestral *V. hybanthoides* populations disperse across continuous mountain barriers to colonize geographically isolated danxia habitats in Danxiashan and Pingshi? (2) Does contemporary or historical inter‐population gene flow persist between these two disjunct regions, or has long‐term geographic isolation driven complete genetic divergence? (3) How have Quaternary climatic oscillations shaped the current population genetic structure and local adaptive patterns of *V. hybanthoides*? This study provides empirical insights into the microevolutionary processes of narrow endemic plants in fragmented danxia landscapes and offers a scientific reference for the conservation management of danxia endemic biodiversity.

## 2 Materials and Methods

### 2.1 Samples Collection

**FIGURE 1:**
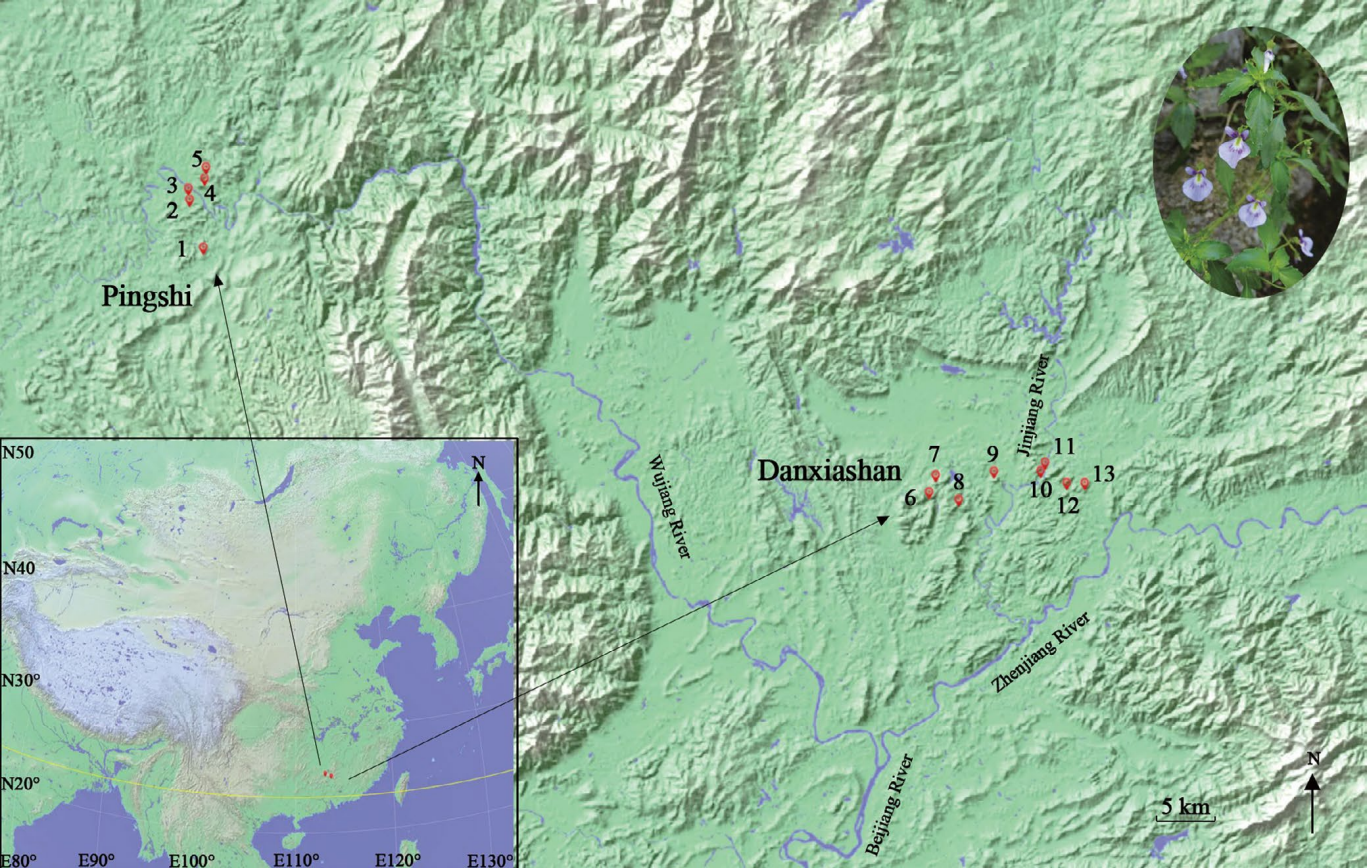
Geographic distribution of sampling sites for *Viola hybanthoides* in Guangdong, China. Populations are located in two danxia landscape regions: Pingshi (populations P1–P5) and Mount Danxiashan (populations P6–P13). The inset map shows the geographic location of the study area in China, and the photograph depicts the flowers and leaves of *V. hybanthoides*.

In this study, we set each independent isolated hilltop cliff habitat as one independent sampling population. A total of 13 populations of *V. hybanthoides* were sampled, comprising 5 populations from Pingshi and 8 from Danxiashan. Field sampling locations were recorded using a Garmin GPS unit (GPSMAP 62sc, Shanghai), and the corresponding geographical coordinates are provided in Table 1 and Figure 1. Fresh leaves were dried and stored in sealed plastic bags with silica gel. Voucher specimens for all populations have been deposited in the Herbarium of Sun Yat‐sen University, China (SYSU).

**TABLE 1:** Geographical locations and genetic diversity of sampled populations of *Viola hybanthoides*.

ID	N	Geographic location	Latitude and longitude	*H*_*O*_	*H*_*E*_	*π*	*F*_*IS*_
Pingshi, Guangdong, China							
P1	15	Tiandingshan	N25°14′13.50″, E113°02′47.39″	0.055 ± 0.001	0.090 ± 0.001	0.095 ± 0.002	0.110
P2	14	Zhichong	N25°16′25.90″, E113°02′21.06″	0.054 ± 0.001	0.094 ± 0.001	0.099 ± 0.001	0.118
P3	13	Lingshiba	N25°16′56.11″, E113°02′20.83″	0.052 ± 0.001	0.077 ± 0.001	0.081 ± 0.001	0.078
P4	5	Jinjiling	N25°17′20.88″, E113°03′11.92″	0.017 ± 0.001	0.011 ± 0.001	0.012 ± 0.001	−0.009
P5	15	Jinjiling Houshan	N25°17′50.85″, E113°03′19.25″	0.048 ± 0.001	0.113 ± 0.001	0.118 ± 0.001	0.172
Mount Danxiashan, Guangdong, China							
P6	15	Yanyan	N25°00′52.53″, E113°38′24.53″	0.037 ± 0.001	0.061 ± 0.001	0.064 ± 0.001	0.071
P7	11	Bianzhai	N25°01′40.60″, E113°38′45.96″	0.042 ± 0.001	0.053 ± 0.001	0.057 ± 0.001	0.035
P8	13	Bazhai	N25°00′26.19″, E113°39′54.05″	0.051 ± 0.001	0.058 ± 0.001	0.061 ± 0.001	0.029
P9	7	Zimeifeng	N25°01′41.79″, E113°41′43.59″	0.012 ± 0.002	0.022 ± 0.001	0.026 ± 0.001	0.027
P10	11	Xianglonghu	N25°01′35.36″, E113°44′05.73″	0.033 ± 0.001	0.042 ± 0.001	0.045 ± 0.001	0.031
P11	13	Yanjiangbudao	N25°01′59.22″, E113°44′19.81″	0.039 ± 0.001	0.044 ± 0.001	0.047 ± 0.001	0.024
P12	11	Pingfengzhai	N25°00′56.11″, E113°45′24.60″	0.021 ± 0.001	0.059 ± 0.001	0.064 ± 0.001	0.106
P13	13	Jiucaizhai	N25°00′53.00″, E113°46′19.41″	0.020 ± 0.001	0.036 ± 0.001	0.040 ± 0.001	0.048

### 2.2 DNA Extraction, Sequencing, and Plastid Genome Assembly

For the majority of populations, we collected 1–2 individuals at random. Total genomic DNA of high molecular weight and integrity was isolated using the TIANamp Genomic DNA Kit (TIANGEN, Beijing) following the manufacturer's protocol. Paired‐end sequencing with 150 bp read length (PE150) was performed on an Illumina X‐Ten platform. Raw reads were quality‐filtered and adapter‐trimmed using fastp v0.23.2 (Chen et al. 2018). For each sample, approximately 6 Gb of clean data were retained and subsequently assembled into circular plastid genomes using NOVOPlasty v2.7.2 (Dierckxsens et al. 2017). The plastid genome and the *rbcL* gene of *Viola japonica* (GenBank accession: MZ151699.1) were used as the reference and seed, respectively.

### 2.3 Phylogenetic Inference Based on Plastid Genomes

The 19 newly assembled plastid genomes, together with 68 publicly available plastid genomes of *Viola* and 2 plastid genomes of *Scolopia* downloaded from the NCBI nucleotide database, were aligned using MAFFT v7.407 (Katoh and Standley 2013). The alignment was manually inspected and adjusted in MEGA‐X (Kumar et al. 2018). Ambiguous sites and gaps were removed prior to phylogenetic analysis. A maximum likelihood (ML) tree was reconstructed in IQ‐TREE v1.6.8 (Nguyen et al. 2015) with the best‐fit substitution model selected using the Bayesian information criterion (BIC).

### 2.4 RAD Sequencing and Data Processing

Genomic DNA extraction, ddRAD‐seq library preparation, and Illumina sequencing were conducted by JieRui BioScience Co. Ltd. (Guangzhou, China) following the standard protocol described in Chen et al. (2021). Raw reads were processed using Stacks v2.55 (Catchen et al. 2011). The pipeline began by demultiplexing RAD tags with process_radtags, discarding samples with fewer than 800,000 retained reads. Optimal parameters were determined using 15 samples, testing M (mismatches within samples) from 1 to 9 with n (mismatches between samples) equal to M. All samples were then processed with the optimal M and n values. The populations module was subsequently used to filter the data (—min‐maf 0.05, —max‐obs‐het 0.8, ‐r 0.8, ‐p 7) for most following analyses (dataset A), while retaining one random SNP per locus for ADMIXTURE analysis. VCF files were converted to other formats using PGDSpider v2.1.1.5 (Lischer and Excoffier 2012). Finally, Tajima's D was calculated in 3000 bp non‐overlapping windows using VCFtools 0.1.17 (Danecek et al. 2011). All loci exhibited Tajima's D values between −1.795 and 2.052, consistent with neutral evolution (Tajima 1989), and were retained for downstream analysis.

### 2.5 Population Data Analyses

Population genetic analyses were conducted on the filtered VCF file. Principal component analysis (PCA) was performed using PLINK v1.90 (Chang et al. 2015) after converting the data to binary PLINK format, and the resulting scatter plots were generated with custom Python scripts (Python Software Foundation 2023) and R v4.0.4 (R Core Team 2021). Bayesian clustering was implemented in ADMIXTURE v1.3.0 (Alexander et al. 2009) with the number of clusters (*K*) ranging from 1 to 13; the optimal *K* was determined based on the cross‐validation (CV) procedure. A maximum likelihood (ML) phylogenetic tree was then constructed from the same VCF file, which was first converted to PHYLIP format using the vcf2phylip.py script (https://github.com/edgardomortiz/vcf2phylip/releases) and then analyzed in IQ‐TREE using the parameters “‐m MFC+ASC –alrt 2000”. Pairwise *F*_*ST*_ values were calculated with VCFtools, and correlation between genetic distance *F*_*ST*_/(1‐*F*_*ST*_) and geographic distance (ln) was tested using a Mantel test in GenAlEx v6.5 (Peakall and Smouse 2012; Diniz‐Filho et al. 2013). Finally, the 13 populations were grouped into three regional clusters: PS (P1–P5), DXW (P6–P8) and DXE (P10–P13), and AMOVA was performed to partition genetic variance among regions, among populations within regions, and within populations using Arlequin 3.5.2.2 (Excoffier and Lischer 2010).

### 2.6 Historical Demographic Analyses

Historical demographic analyses were performed to reconstruct the temporal population dynamics and divergence history of *V. hybanthoides*. One‐dimensional folded site‐frequency spectra (1D‐SFS) were generated from the filtered VCF dataset using easySFS (https://github.com/isaacovercast/easySFS) for subsequent demographic inference implemented in Stairway Plot 2 (Liu and Fu 2015). All individuals were assigned into three regional population clusters: PS (P2–P5), DXW (P6–P8), and DXE (P10–P13). To retain the maximum number of segregating sites while minimizing missing data bias, SFS projection sizes were set to 38, 40, and 34 gene copies for the PS, DXW, and DXE clusters, respectively. To convert scaled coalescent time into absolute chronological time, we adopted a per‐site per‐generation mutation rate of 6.5 × 10^−9^ (Ossowski et al. 2010) and a one‐year generation time. All remaining parameters were kept as default throughout the Stairway Plot 2 analyses.

To further clarify the genetic divergence between Pingshi and Danxiashan populations and to unravel the evolutionary origin of the admixed P1 population, we conducted demographic model selection using fastsimcoal2 (Excoffier et al. 2021). Individuals were grouped into three major genetic pools: P1 (putative admixed populations in Pingshi), P2–P5 (non‐admixed Pingshi populations), and P6–P13 (Danxiashan populations), with SFS projection sizes standardized to 22, 50, and 64 gene copies, respectively. Five alternative demographic models (M1–M5) were constructed and compared to test competing divergence and admixture scenarios. M1: Initial divergence occurred between Pingshi and Danxiashan lineages at time T1, followed by the derivation of P1 from Pingshi at time T0. M2: Pingshi and Danxiashan lineages initially diverged at T1, with P1 subsequently originating from Danxiashan at T0. M3: Pingshi and Danxiashan lineages split at the deeper time point T2; P1 diverged from Danxiashan at T1, followed by unidirectional gene flow from Pingshi into P1 at T0. M4: Lineage divergence between Pingshi and Danxiashan occurred at T2; P1 split from Pingshi at T1, followed by unidirectional gene flow from Danxiashan into P1 at T0. M5: Following basal divergence between Pingshi and Danxiashan lineages at T1, a single pulse of bidirectional gene flow at T0 gave rise to the admixed P1 population.

For each model, 50,000 expected SFS replicates were simulated, and 40 expectation–maximization cycles were executed per simulation run. Each model was independently repeated 50 times to avoid local likelihood optima. For each model iteration, the replicate with the highest composite likelihood was retained for downstream comparison. Model performance was evaluated using the Akaike Information Criterion (AIC), which accounts for both model likelihood and structural complexity. The optimal demographic model was determined based on delta AIC (ΔAIC) values.

## 3 Results

### 3.1 Shallow Genome Sequencing and Phylogenetic Analysis Based on Plastid Genome

**FIGURE 2:**
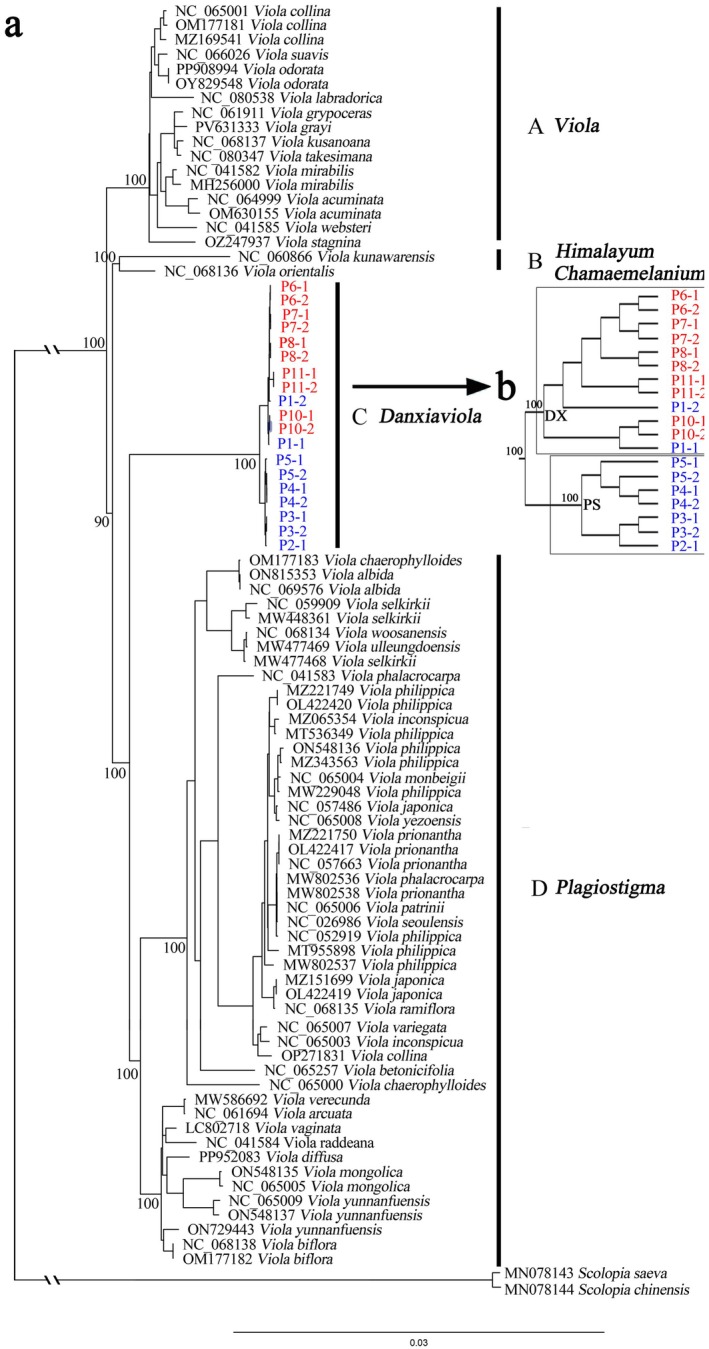
Maximum likelihood phylogeny based on complete chloroplast genomes of *Viola hybanthoides* and related species. (a) The full phylogram, with ML bootstrap support values shown near the nodes; individuals from Pingshi are marked in blue, and those from Danxiashan are marked in red. (b) Cladogram of the *Danxiaviola* clade, with branch lengths removed to clarify topological relationships (branch lengths are not proportional to genetic distance).

From approximately 6 Gb of raw data per sample, we successfully assembled and circularized 19 plastid genomes, which ranged in length from 156,637 to 156,696 bp and have been deposited in the NCBI nucleotide database under accession numbers PX991862–PX991880. A phylogenetic tree constructed from these 19 newly assembled genomes together with 68 published plastid genomes revealed that all examined *Viola* species were subdivided into four well‐supported clades (A–D; Figure 2), corresponding to sect. *Viola* (A), *Himalayum* and *Chamaemelanium* (B), *Danxiaviola* (C), and *Plagiostigma* (D). Within clade C, all *V. hybanthoides* samples formed a distinct mono‐group that could be further divided into two subclades: PS, comprising samples from populations P2–P5 collected in Pingshi, and DX, consisting of all samples from Danxiashan and two samples from population P1 in Pingshi.

### 3.2 Genetic Diversity Estimation

A total of 13 populations of *V. hybanthoides* were analyzed, including 5 from Pingshi and 8 from Danxiashan. Following data filtration, 14,215 loci containing 41,337 variant sites were retained for dataset A; for dataset R, a single SNP was randomly selected per locus, with 10,042 variant sites retained after this filtering step.

Based on dataset A, the Pingshi populations had a mean observed heterozygosity (*H*_*O*_) of 0.046 (range: 0.017–0.055), a mean expected heterozygosity (*H*_*E*_) of 0.077 (range: 0.011–0.113), a mean nucleotide diversity (π) of 0.081 (range: 0.012–0.118), and a mean inbreeding coefficient (*F*_*IS*_) of 0.094 (range: −0.009–0.172). Notably, population P4 exhibited markedly lower values across all these genetic parameters, which is likely attributable to its small sample size (only 5 individuals). For the Danxiashan populations, the corresponding mean values were 0.025 for *H*_*O*_ (range: 0.012–0.051), 0.041 for *H*_*E*_ (range: 0.022–0.061), 0.044 for π (range: 0.026–0.064), and 0.047 for *F*_*IS*_ (range: 0.024–0.106). Similarly, population P9 showed reduced genetic diversity indices across all measures, potentially due to its limited sample size of 7 individuals.

### 3.3 Population Structure

**FIGURE 3:**
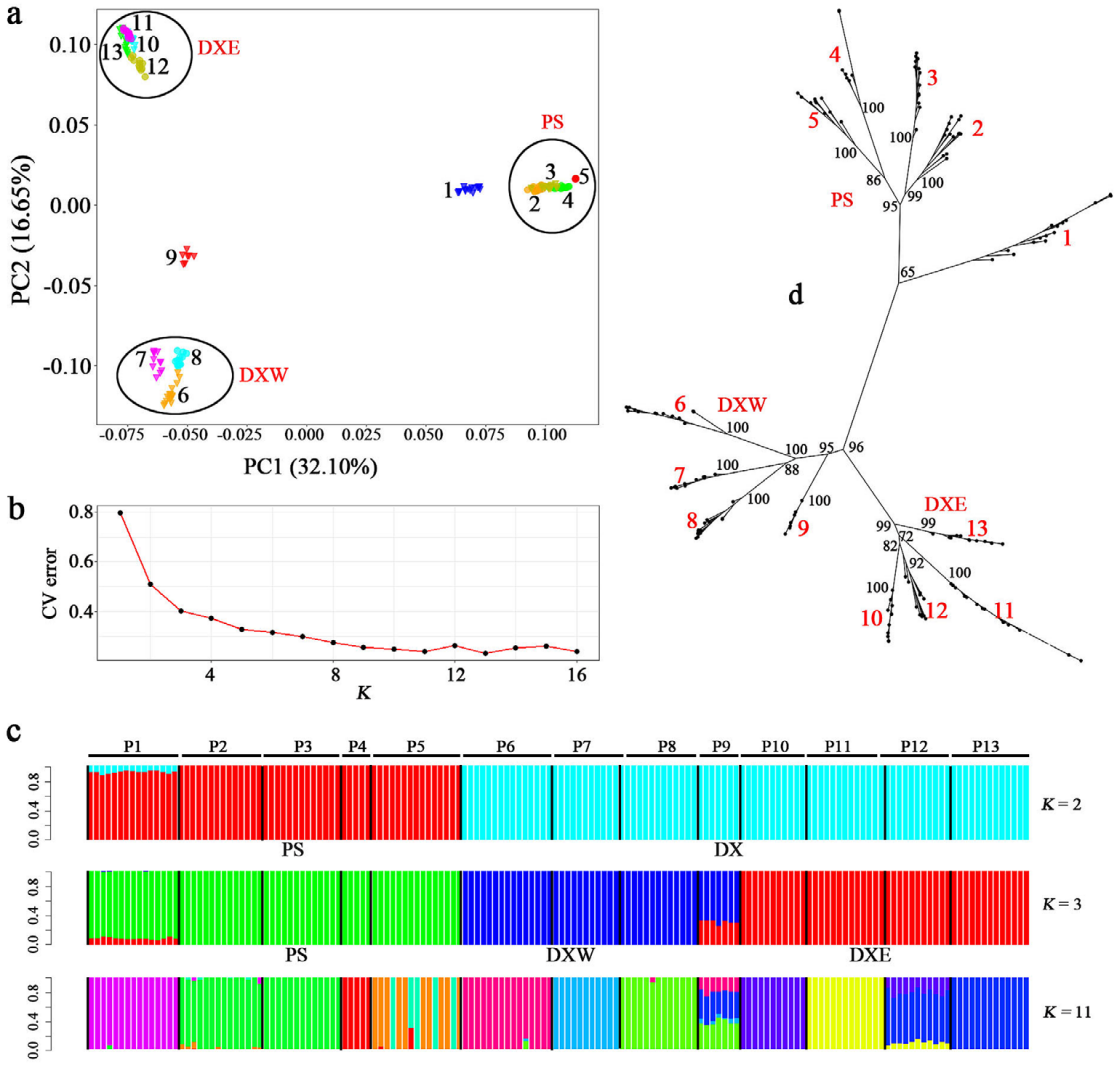
Population genetic structure of 13 populations (156 individuals) of *Viola hybanthoides* based on ddRAD‐seq data. (a) Principal component analysis (PCA), with individuals color‐coded by population. (b) Cross‐validation (CV) error plot from ADMIXTURE analysis, used to identify the optimal number of genetic clusters (*K*). (c) ADMIXTURE bar plots for *K* = 2, 3, and 11. Each vertical bar represents one individual, and colors correspond to inferred genetic ancestry components. (d) Maximum likelihood (ML) phylogenetic tree based on ddRAD‐seq data. Bootstrap support values for major clades are shown at the nodes. labels 1–13 correspond to populations P1–P13.

Principal component analysis (PCA) based on genome‐wide ddRAD‐seq SNPs partitioned genetic variation across all sampled individuals. The first two principal components (PC1 and PC2) accounted for 32.10% and 16.65% of total genomic variance, respectively. PC1 sharply differentiated two major regional genetic clusters: Pingshi cluster (PS, populations P2–P5) and Danxiashan cluster (DX, populations P6–P13). Notably, all individuals in population P1 from Pingshi occupied an intermediate position on the PCA plot and lay closer to the PS cluster. Within Danxiashan cluster, PC2 further resolved distinct western (DXW, P6–P8) and eastern (DXE, P10–P13) subclusters. Geographically central population P9 fell midway between the DXW and DXE subgroups on the PCA ordination plot (Figure 3a).

ADMIXTURE clustering corroborated the regional genetic partitioning revealed by PCA and further resolved fine‐scale ancestral components across the 13 *V. hybanthoides* populations. Although the absolute minimum cross‐validation (CV) error occurred at *K* = 13, a distinct elbow point in the CV curve at *K* = 11 indicated this value represented the biologically meaningful optimal number of genetic clusters (Figure 3b). At *K* = 2, the two ancestral gene pools corresponded well to the two broad regional clusters defined by PC1: populations P2–P5 exclusively constituted the PS gene pool, while all Danxiashan populations belonging to the DX gene pool. Consistent with its intermediate position in the PCA plot, population P1 harbored mixed ancestry derived from both PS and DXS, with PS as the dominant genetic component. At *K* = 3, populations in Danxiashan split into two gene pools: DXW (P6–P8) and DXE (P10–P13). The geographically transitional population P9 exhibited shared ancestry from DXW and DXE. When *K* increased to 11, five independent gene pools were identified in the Pingshi and six in Danxiashan, indicating pronounced fine‐scale genetic differentiation among populations (Figure 3c).

A phylogenetic tree constructed from ddRAD‐seq data (Figure 3d) revealed that all individuals from the same population formed monophyletic clades. All Pingshi populations constituted clade PS, with P1 located at the basal position. Similarly, all Danxiashan populations formed clade DX. Within this clade, populations P6–P8 and the central population P9 formed subclade DXW with P9 at its base, whereas the four eastern populations (P10–P13) formed another subclade DXE (Figure 3d).

### 3.4 Population Differentiation

Pairwise *F*_*ST*_ values (Table S1) illustrated clear hierarchical patterns of genetic divergence. Within local geographic regions, genetic differentiation among populations was generally moderate and relatively low. In Pingshi (P1–P5), pairwise *F*_*ST*_ ranged from 0.198 to 0.487 with a mean value of 0.329. The western Danxiashan populations (P6–P8) had slightly higher within‐group differentiation, with *F*_*ST*_ varying from 0.371 to 0.403 (mean = 0.383). By comparison, eastern Danxiashan populations (P10–P13) showed a wider *F*_*ST*_ range of 0.266–0.430 and a mean of 0.344. Genetic divergence increased markedly between different sub‐regions. Moderately high differentiation was detected between western and eastern populations within Danxiashan, with pairwise *F*_*ST*_ ranging from 0.440 to 0.566 (mean = 0.515). Notably, the strongest genetic differentiation occurred between the Pingshi and Danxiashan, where inter‐regional *F*_*ST*_ values spanned 0.455–0.797 and reached a high mean of 0.573.

**FIGURE 4:**
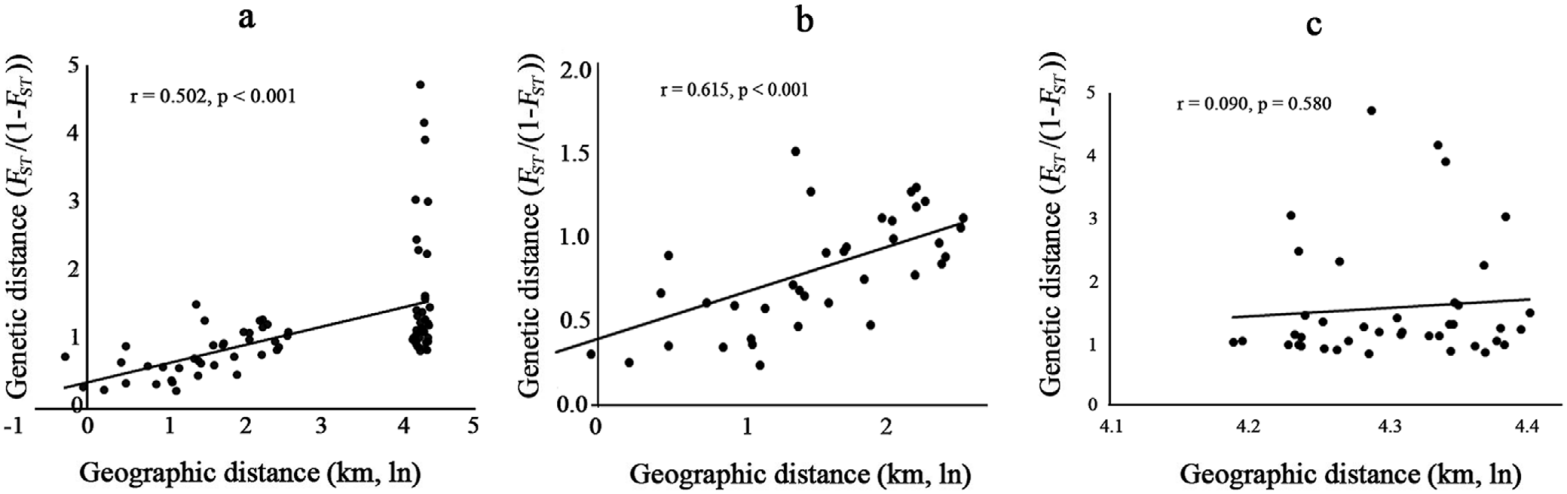
Isolation‐by‐distance (IBD) patterns in *Viola hybanthoides* populations. Correlations between genetic distance, calculated as *F*_*ST*_/(1‐*F*_*ST*_), and natural‐log‐transformed geographic distance (km) are shown for (a) all population pairs across both regions, (b) population pairs within the same region (Pingshi or Mount Danxiashan), and (c) population pairs between the two regions (Pingshi vs. Mount Danxiashan). Pearson correlation coefficients (*r*) and corresponding *p* values are presented in each panel.

We further tested the isolation by distance (IBD) pattern by correlating genetic distance *F*_*ST*_ / (1‐ *F*_*ST*_) with log‐transformed geographic distance. A significant positive IBD signal was observed across all population pairs (*r* = 0.502, *p* < 0.001, Figure 4a). When datasets were grouped by geographic range, strong significant IBD remained valid for population pairs within Pingshi or within Danxiashan (*r* = 0.615, *p* < 0.001, Figure 4b). However, this correlation disappeared completely between populations originating from the two independent major regions (*r* = 0.09, *p* = 0.580, Figure 4c).

Analysis of molecular variance (AMOVA; Table 2) further confirmed this strong regional genetic structure. The largest proportion of total genetic variation (45.44%) was attributed to divergence among major geographic regions, followed by variation among populations within each region (41.93%). In contrast, genetic variation residing within individual populations only accounted for 12.63% of the total variance.

**TABLE 2:** Analysis of molecular variance for ddRAD‐seq data of *Viola hybanthoides*.

Source of variation	d.f.	Sum of squares	Variance components	Percentage of variation	Fixation Indices
Among groups	2	206.881	1.631	45.44	*F*_*CT*_: 0.45441
Among populations Within groups	10	182.329	1.505	41.93	*F*_*SC*_: 0.76853
Within populations	143	64.835	0.453	12.63	*F*_*ST*_: 0.87371
Total	155	454.045	3.590	100	

### 3.5 Demographic History

**FIGURE 5:**
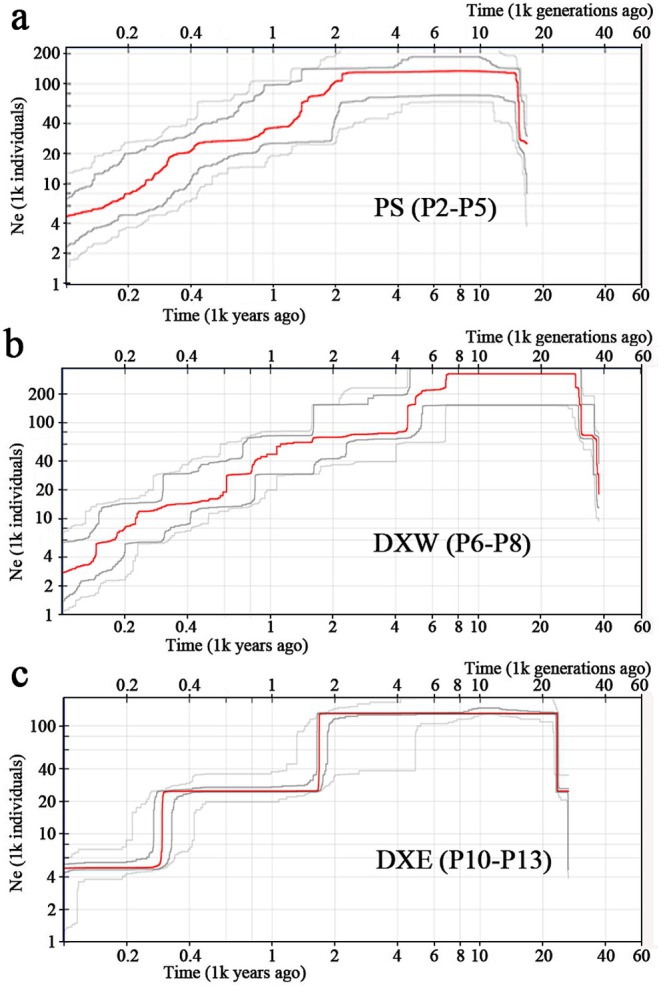
Demographic history of *Viola hybanthoides* populations inferred by stairway plot analysis. (a) Pingshi (PS: P2–P5); (b) West of Mount Danxiashan (DXW: P6–P8); (c) East of Mount Danxiashan (DXE: P9–P13). Red lines = median estimates of effective population size *N*_*e*_; gray lines = 95% confidence intervals.

Demographic history reconstruction revealed distinct effective population size *N*_*e*_ dynamics across the three PCA‐defined genetic clusters (Figure 5). The PS cluster (P2–P5) experienced long‐term population expansion and subsequently underwent a recent severe bottleneck. Its *N*_*e*_ rose steadily from around 20 thousand years ago (kya) and reached a historical peak before declining sharply since 2 kya to a relatively small modern population size (Figure 5a). The DXW subcluster (P6–P8) shared a similar expansion—contraction trend. It started rapid population growth as early as 40 kya and achieved the largest historical population size among all three clusters, followed by sustained population decline since 8 kya. To date, DXW has the smallest contemporary effective population size (Figure 5b). In comparison, the DXE subcluster (P10–P13) began obvious population expansion later at approximately 30 kya, maintained a stable moderate population scale for a long period, and also experienced population reduction starting from 2 kya. Its current *N*_*e*_ is slightly larger than that of the western group (Figure 5c).

### 3.6 Gene Flow Between Pingshi and Danxiashan

**FIGURE 6:**
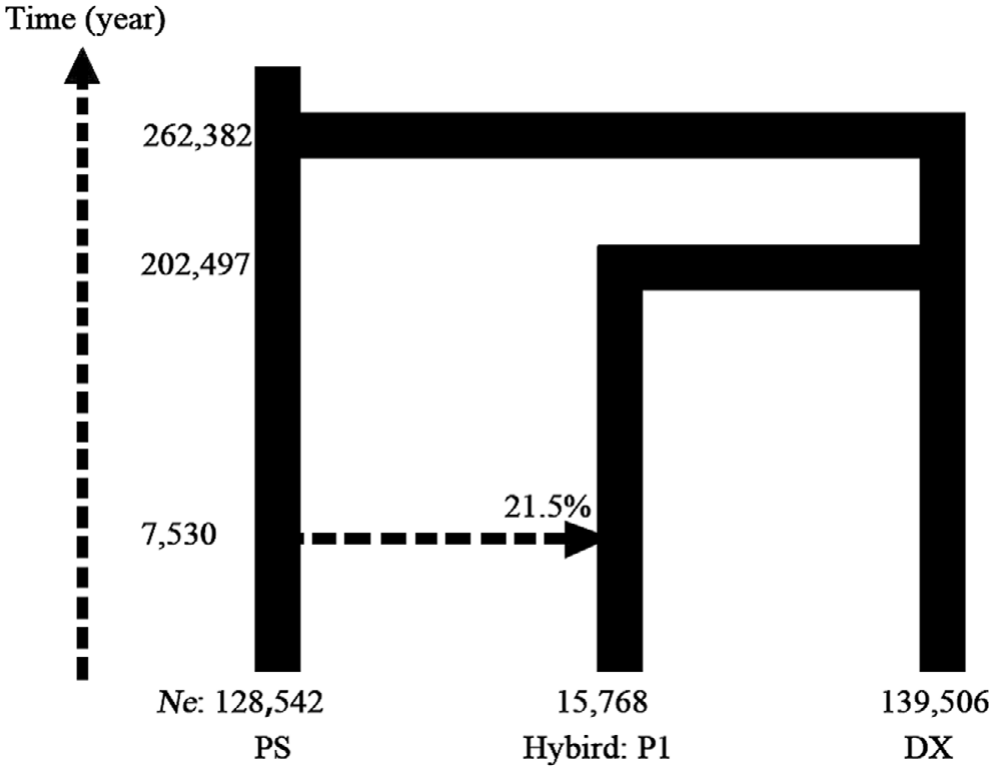
Schematic diagram illustrating the demographic simulation of population P1, depicting its hybrid origin via admixture between the Pingshi (PS) and Mount Danxiashan (DX) lineages. The plot shows divergence times (years before present), effective population sizes (*N*_*e*_), and the estimated proportion of genetic admixture (21.5%) from the PS lineage into P1.

Based on the comparative analysis of five demographic models using fastsimcoal2, Model M3 was identified as the optimal model (Table S2) for describing the evolutionary relationships among the population P1, the PS cluster (P2–P5), and the DX cluster (P6–P13). This selected model provides an estimated timeline of divergence and admixture events: the PS populations diverged from the DX populations approximately 250,000 years ago; subsequently, around 200,000 years ago, the DX populations underwent further diversification, potentially splitting into sub‐lineages or initiating expansion; around 8647 years ago, the expanding DX lineage reached the geographical vicinity of the PS populations, leading to gene flow between them and resulting in the formation of the hybrid population (Hybrid: P1). Specifically, 21.5% of the genetic material in the P1 hybrid population is derived from the PS parental populations (Figure 6).

## 4 Discussion

### 4.1 Low Genetic Diversity and Mating System

Our analyses reveal that *V. hybanthoides* exhibits a low level of genetic diversity (*H*_*E*_ = 0.059 ± 0.028), which is lower than that reported for its endangered congener *V. grayi* (average *H*_*E*_ = 0.142 based on SSR markers; Hirai et al. 2012) and several more widespread *Viola* species, including *V. pubifolia* and *V. palustris* (*H*_*E*_ = 0.28 based on RAD‐seq data; Żabicka et al. 2023) as well as *V. pubescens* (*H*_*E*_ = 0.23 based on allozyme data; Culley and Grubb 2003). In contrast, its genetic diversity is more comparable to that of other Danxiashan endemics, such as *F. danxiaensis* (*H*_*E*_ = 0.116 based on ddRAD‐seq data; Chen et al. 2024) and *P. danxiaensis* (*H*_*E*_ = 0.063 based on ddRAD‐seq data; Chen et al. 2021).

The low within‐population genetic diversity of *V. hybanthoides* could be shaped by the combined effects of the unique danxia landscape, the intrinsic biological traits of the species, past climate changes, and anthropogenic disturbance. Firstly, the unique geomorphological properties of danxia landscape create inherently fragmented habitats and strong geographical isolation. Discrete cliff habitats are isolated and surrounded by heterogeneous non‐danxia matrix environments, forming solid natural geographical barriers that physically separate different wild populations (Haig et al. 2000; Peng et al. 2011). Further, most species of the genus *Viola* only achieve pollen and seed dispersal within a narrow radius of 3 m (Beattie 1976), and the seeds of *V. hybanthoides* rely exclusively on short‐distance ballistic ejection for propagation (personal observations). This extremely limited dispersal capacity of *V. hybanthoides* makes it difficult to break through the above geographical isolation and establish effective interpopulation gene flow. Additionally, the long‐term historical population dynamics accelerated the continuous loss of genetic variation. Demographic history showed that *V. hybanthoides* thrived under glacial climatic conditions, whereas it declined sharply over the past ten thousand years in west Danxiashan (Figure 5). It was possibly that post‐glacial climate warming promoted the rapid expansion of subtropical forests, which greatly compressed and fragmented its suitable cliff habitats (Wang, Xu, et al. 2017). Beyond natural climatic changes, the rise and continuous development of human civilization over the past nearly 2000 years has further intensified regional habitat fragmentation (Ellis and Ramankutty 2008), leading to a further reduction in the effective population size of this species in Pingshi and eastern Danxiashan (Figure 5). At present, surviving populations are only confined to scattered cliff refugia, which further depletes the original genetic variation of populations. The phylogenetic tree based on ddRAD‐seq data showed that individuals from each natural population clustered into an independent monophyletic clade (Figure 3d), confirming that extant cliff populations were derived from a small number of founder individuals. Such a narrow genetic foundation at the initial colonization stage laid an inherent constraint on the improvement of intrapopulation genetic diversity (Sendell‐Price et al. 2021). In summary, the innate fragmentation of danxia landscape, weak species dispersal ability, post‐glacial population contraction and human‐mediated habitat disturbance jointly restrict interpopulation gene flow and promote the prevalence of founder events (Haig et al. 2000). Together with strong genetic drift and weak heterozygote deficiency (*F*_*IS*_ averagely 0.064, Table 1) prevailing in small isolated populations, these multiple driving factors maintain the persistently low within‐population genetic diversity of *V. hybanthoides*. Low genetic diversity in turn weakens the species' environmental adaptability and range expansion capacity, forming a negative feedback loop that severely hinders population recovery and restoration (Zapata et al. 2020). Our results also support the classic evolutionary theory that habitat‐specialized endemic species inhabiting isolated and fragmented habitats generally exhibit reduced within‐population genetic diversity (Frankham et al. 2010).

### 4.2 Spatial Genetic Structure Driven by Quaternary Climatic Fluctuations and Landscape Barriers

ADMIXTURE and PCA analyses revealed significant inter‐regional genetic differentiation among *V. hybanthoides* populations from the two geographically isolated danxia landscapes. At *K* = 2, all individuals were clearly assigned to two distinct ancestral gene pools: PS exclusively comprised individuals from all Pingshi populations, while DX covered all populations from Danxiashan (Figure 3c), which was highly consistent with PCA analysis (Figure 3a). Consistent with these clustering patterns, the AMOVA analysis showed that 45.44% of the total genetic variation was attributed to inter‐regional divergence, representing the predominant source of genomic differentiation in this species (Table 2). Demographic reconstruction results revealed that *V. hybanthoides* underwent a pronounced population expansion ~20–40 kya during the Last Glacial Period (Figure 5). Combined with the spatiotemporal dynamics of local landscape evolution, we speculate that glacial cooling inhibited the development of subtropical evergreen forests and exposed extensive, interconnected red sandstone cliff outcrops, consequently forming continuous suitable habitats for *V. hybanthoides* across the landscape (Wang, Xu, et al. 2017). Subsequent post‐glacial warming during the Holocene promoted the rapid recovery and expansion of forest vegetation. The regenerated forests occupied valley bottoms and fragmented the formerly contiguous danxia cliff habitats. Such landscape transformation confined *V. hybanthoides* populations to two isolated interglacial refugia in Pingshi and Danxiashan, which is consistent with previous phylogeographic studies on Danxiashan‐endemic plant species (Peng 2020; Chen et al. 2024).

In addition to landscape isolation, the genetic divergence of *V. hybanthoides* is further reinforced by its limited dispersal capacity, a common trait among *Viola* species. Specifically, pollination relies on local bee or beefly communities with short‐distance foraging ranges, while seed dispersal depends on ballistic ejection, which restricts long‐distance population connectivity (Beattie 1976; Fan et al. 2015). Isolation‐by‐distance (IBD) analyses further supported this dispersal limitation pattern: significant positive correlations between genetic and geographic distances were detected within individual contiguous danxia outcrops, whereas no IBD signal was observed between the two geographically separated mountain regions (Figure 4). This heterogeneous IBD pattern indicates that *V. hybanthoides* can maintain limited gene flow within locally continuous danxia habitats but is unable to establish effective inter‐regional genetic exchange across the intervening unsuitable matrix of forests, farmlands, and low hills. Long‐term geographic isolation, coupled with founder effects and persistent genetic drift, has driven the gradual accumulation of substantial genetic differentiation between the two refugial lineages (Storfer et al. 2007). Overall, the contemporary spatial genetic structure of *V. hybanthoides* is shaped by the interactive effects of Quaternary climatic oscillations, post‐glacial habitat fragmentation, modern landscape ecological barriers, and the species' intrinsic low dispersal ability.

The Jinjiang River splits Danxiashan into eastern and western parts, which can impede inter‐population gene flow of numerous plant species across the river. Multiple cases have confirmed that rivers can act as effective barriers to plant gene flow (Geng et al. 2015; Cazé et al. 2016). Previous studies on *F. danxiaensis* have verified the isolating effect of the Jinjiang River, though genetic introgression from populations on the opposite bank is detectable in most local populations (Chen et al. 2024). The present study on *V. hybanthoides* further exhibits a more distinct east–west genetic differentiation pattern. Both PCA and ADMIXTURE analyses demonstrated that all sampled populations in Danxiashan were clearly separated into two genetic subclusters, namely DXW and DXE. Most *V. hybanthoides* populations retain genetically homogeneous gene pools, while only one population (P9) located close to the Jinjiang River shows admixed genetic compositions (*K* = 3, Figure 3c). This suggests that *V. hybanthoides* has weaker dispersal ability than *F. danxiaensis*. Accordingly, the river exhibits a stronger isolating effect, leading to much more pronounced genetic differentiation among its populations.

### 4.3 Secondary Contact Among Isolated Danxia Landscapes

A key unresolved scientific issue lies in whether historical gene flow occurred between the two geographically disjunct danxia landscapes, namely Pingshi and Danxiashan, and how *V. hybanthoides* managed to disperse across such spatial gaps. Our multi‐level genomic data firmly verify the occurrence of inter‐regional secondary contact between populations from the Pingshi and Danxiashan. ADMIXTURE clustering results indicated that population P1 harbors admixed nuclear genetic components derived from both Danxiashan and local resident populations in Pingshi (Figure 3c). Further, plastid phylogenetic analyses revealed that the two plastid genomes randomly selected from the population P1 are nested within the DX clade (Figure 2). Consistent with both nuclear and cytoplasmic genetic evidence, demographic simulations conducted via fastsimcoal2 identified the admixture model as the optimal evolutionary scenario. This model inferred unidirectional gene flow from Danxiashan into ancestral Pingshi resident populations approximately 9 kya, which eventually facilitated the formation of the admixed P1 lineage.

Combining nuclear and plastid genetic evidence, demographic reconstruction results and paleoclimatic dynamics, we herein propose a glacial expansion‐admixture hypothesis to explain this cross‐regional genetic exchange. During the Quaternary glaciations, climate cooling triggered widespread range contraction of subtropical evergreen forests, exposing large areas of interconnected red cliff habitats across northern Guangdong (Kershaw et al. 2001). This habitat continuity promoted substantial population expansion of *V. hybanthoides*, enabling its range expansion from Danxiashan to the Pingshi (Figure 5). After successful colonization, immigrants originating from Danxiashan interbred with local resident populations in Pingshi, ultimately forming the genetically stable admixed population P1. With climate warming at the beginning of the Holocene epoch, subtropical evergreen forests reoccupied valley lowlands, fragmenting the once continuously distributed cliff habitats and resulting in the long‐term stable geographical isolation between Pingshi and Danxiashan observed at present (Wang, Xu, et al. 2017; Peng et al. 2018; Chen et al. 2024).

Similar patterns of historical inter‐landscape gene flow have also been reported in *F. danxiaensis*, in which nuclear genetic introgression was detected from danxia landscape to adjacent karst habitat, whereas no long‐distance seed‐mediated plastid gene flow was observed in that system (Chen et al. 2024). In contrast, our study captured unambiguous signatures of both nuclear and plastid gene flow across the approximately 60 km mountain matrix separating Pingshi and Danxiashan. These solid genomic evidences strongly suggest that the two currently isolated danxia landscapes served as interconnected glacial refugia for *V. hybanthoides* (Hampe et al. 2013), which were functionally linked by continuous cliff habitats that were widely available during cold glacial periods.

### 4.4 Conservation Implications

Danxia landscapes are widely scattered across subtropical southern China, yet most individual danxia patches are small (< 10 km^2^) and isolated by forested mountain matrices, rarely sustaining large, genetically stable endemic plant populations or serving as long‐term climatic refugia (Peng 2020). In contrast, the two geographically separated danxia landscapes hosting *V. hybanthoides* (Pingshi and Danxiashan) represent exceptional large red‐bed habitat complexes: Danxiashan spans 292 km^2^ and contains over 680 discrete red cliff outcrops, while Pingshi harbors two independent large danxia blocks covering 60 km^2^ and 17 km^2^, respectively (Peng et al. 2011; Peng 2020). These two landscape units act as the sole interglacial refugia for this narrow endemic *Viola* species and host more than 20 danxia‐restricted vascular plant species with high conservation value in Danxiashan (Fan et al. 2022). Our population genomic results reveal multiple severe threats to the long‐term persistence of *V. hybanthoides*, which necessitates targeted, hierarchical conservation strategies tailored to its unique genetic structure, life‐history traits, and fragmented cliff habitats.

Our study identifies four key intrinsic and extrinsic risk factors threatening the survival of *V. hybanthoides*. (1) Extremely low within‐population genetic diversity (mean *H*_*E*_ = 0.058 ± 0.028) across all sampled populations, far lower than widespread congeneric *Viola* species and comparable to critically endangered danxia endemics such as *P. danxiaensis* (Chen et al. 2021). Small, isolated cliff populations suffer persistent genetic drift and founder effects, which continuously erode standing genetic variation and reduce the species' capacity to cope with future climate disturbance, pests, and disease outbreaks (Hanski 2011; Zapata et al. 2020). (2) Extreme genetic fragmentation and strong hierarchical population differentiation. AMOVA results show that 45.44% of total genomic variation segregates between Pingshi and Danxiashan, with an additional 41.93% partitioned among local populations within each landscape. Significant genetic divergence exists not only between the two major refugia separated by a 60 km mountain barrier, but also between Jinjiang‐divided eastern and western subclusters within Danxiashan. Limited natural gene flow restricts genetic rescue between isolated populations (Frankham 2015). (3) Severe dispersal limitation restricts natural population recovery. Unlike many *Viola* species with ant‐mediated secondary seed dispersal, *V. hybanthoides* lacks seed elaiosomes and only disperses seeds 1–2 m via ballistic ejection; its erect rhizomes also lose effective clonal expansion capacity. Short‐distance beefly pollination further limits pollen exchange, making natural habitat connectivity restoration slow and difficult (Beattie 1974, 1976; Ohkawara and Higashi 1994; Fan et al. 2015). (4) Post‐glacial population bottlenecks combined with recent anthropogenic interference. Stairway Plot demographic reconstructions demonstrate that all three major genetic clusters (PS, DXW, DXE) experienced dramatic population contraction after Holocene warming. Valley forests expanded and split continuous glacial cliff habitats into isolated terrestrial islands over the past 10,000 years (Kershaw et al. 2001); recent human activities including trail construction, scenic development, firewood collection and small‐scale farming have further fragmented cliff microhabitats and compressed the species' already narrow distribution range (Harrison et al. 2024).

Based on our multi‐population genomic data, we propose a multi‐tiered integrated conservation framework for *Viola hybanthoides*, covering in situ core population protection, ex situ germplasm preservation, and permanent monitoring plots (National Forestry and Grassland Administration of China 2018; State Administration for Market Regulation and Standardization Administration of China 2025). First of all, prioritize strict in situ protection of genetically representative core populations (Bates 2002). All 13 populations carry unique genetic components and collectively preserve the full species gene pool, but populations with elevated genetic diversity and mixed ancestry require top‐tier conservation priority. Within Pingshi, P1, P2, and P5 exhibit the highest *H*_*E*_ and nucleotide diversity *π*, with P1 serving as the unique natural hybrid lineage recording historical cross‐landscape secondary contact between Pingshi and Danxiashan; this population represents irreplaceable evolutionary material for studying inter‐danxia dispersal and admixture processes. Within Danxiashan, P6, P8, and P12 maintain relatively high genetic diversity and represent the core gene pools of western and eastern subclusters separated by the Jinjiang River. Local forestry and national park management departments should set up permanent fenced protection zones around these six core populations, prohibit trail expansion, cliff mining, and vegetation clearance within a 50 m buffer zone, and regularly remove invasive herbaceous competitors that outcompete *V. hybanthoides* on thin cliff soils. Second, carry out systematic ex situ germplasm conservation and reinforce wild populations through tissue‐culture‐based reintroduction (Sato et al. 1995). Given the species' weak natural recruitment capacity, we recommend building a dedicated germplasm bank for *V. hybanthoides*. Tissue culture protocols should be optimized using rhizome explants collected from all 13 natural populations to preserve full genetic representation, avoiding genetic bottlenecks during clonal propagation. Mass‐produced seedlings should be reintroduced to suitable, currently unoccupied cliff habitats within the two major danxia landscapes to expand overall population size. Third, implement long‐term population dynamic monitoring with multiple indicators. Permanent monitoring plots should be established for all natural populations to track annual individual survival, clonal growth, fruiting success, and seedling recruitment (Antão et al. 2026). Combined with periodic low‐coverage resequencing every 3–5 years, managers can continuously monitor shifts in genetic diversity, inbreeding coefficients, and genetic differentiation to evaluate the effectiveness of conservation measures. Monitoring data will also help predict population responses to future climate warming, which may further reduce suitable dry cliff habitats for this cold‐adapted danxia endemics.

## Author Contributions

**Sufang Chen:** data curation (equal), formal analysis (equal), writing – original draft (equal), writing – review and editing (equal). **Yanshuang Huang:** data curation (equal), formal analysis (equal), validation (equal), visualization (equal). **Zhiyi Xie:** funding acquisition (supporting), investigation (equal). **Jianqiang Guo:** funding acquisition (equal), investigation (equal). **Fang Chen:** funding acquisition (equal), investigation (equal). **Wenbo Liao:** project administration (equal), writing – review and editing (equal). **Qiang Fan:** conceptualization (equal), methodology (equal), project administration (lead), supervision (lead), writing – review and editing (equal).

## Funding

This work was supported by the Project for Establishment of a Long‐term Forest Monitoring Plot System within the Danxiashan National Park Candidate Area (20261504), the Guangdong Provincial Special Research Grant for the Creation of National Parks (2021GJGY034), Introduction, Propagation and Reintroduction Program for Endemic Plants of Danxiashan (SY25GZ040), and the 2024 Guangdong Province Ecological Quality Index (EQI) Monitoring Project (GPCGD241115FG155F).

## Conflicts of Interest

The authors declare no conflicts of interest.

## Supporting information


**Table S1:** Pairwise FST between sampled populations of *Viola hybanthoides*.
**Table S2:** Comparison of five demographic models for verifying inter‐population hybridization between Pingshi and Danxiashan populations of *Viola hybanthoides*. Notes: MaxEstLhood, maximum estimated log‐likelihood; No. of params, number of estimated parameters; AIC, Akaike information criterion; ΔAIC, difference in AIC values relative to the optimal model with the lowest AIC value.

## Data Availability

The plastid genome sequences of our study involved are deposited in GenBank with accession numbers PX991862–PX991880; the raw data of RAD‐seq data was uploaded to GenBank under the BioProject PRJNA1418919.
