# Supplementary material for: Quaternary Climate Oscillations Shape Genetic Diversity and Spatial Structure of Viola hybanthoides (Violaceae) Endemic to Danxia Landscape
Source: Ecol Evol. 2026 Jul 29;16(8):e74043. doi: 10.1002/ece3.74043 (PMC13416757; doi:10.1002/ece3.74043)
Supplement: Supplementary file 1 — Table S1: Pairwise FST between sampled populations of Viola hybanthoides. Table S2: Comparison of five demographic models for verifying inter‐population hybridization between Pingshi and Danxiashan populations of Viola hybanthoides. Notes: MaxEstLhood, maximum estimated log‐likelihood; No. of params, number of estimated parameters; AIC, Akaike information criterion; ΔAIC, difference in AIC values relative to the optimal model with the lowest AIC value. [file ECE3-16-e74043-s001.docx]

Table S1 Pairwise *F_ST_* between sampled populations of *Viola hybanthoides*

|  | P2 | P3 | P4 | P5 | P6 | P7 | P8 | P9 | P10 | P11 | P12 | P13 |
| --- | --- | --- | --- | --- | --- | --- | --- | --- | --- | --- | --- | --- |
| P1 | 0.324 | 0.384 | 0.487 | 0.328 | 0.508 | 0.501 | 0.485 | 0.475 | 0.531 | 0.541 | 0.466 | 0.489 |
| P2 |  | 0.210 | 0.383 | 0.198 | 0.521 | 0.534 | 0.509 | 0.541 | 0.565 | 0.566 | 0.508 | 0.548 |
| P3 |  |  | 0.474 | 0.260 | 0.574 | 0.590 | 0.558 | 0.585 | 0.616 | 0.622 | 0.555 | 0.597 |
| P4 |  |  |  | 0.239 | 0.712 | 0.753 | 0.698 | 0.826 | 0.807 | 0.797 | 0.693 | 0.752 |
| P5 |  |  |  |  | 0.495 | 0.492 | 0.472 | 0.455 | 0.526 | 0.526 | 0.461 | 0.495 |
| P6 |  |  |  |  |  | 0.403 | 0.376 | 0.481 | 0.543 | 0.550 | 0.472 | 0.529 |
| P7 |  |  |  |  |  |  | 0.371 | 0.478 | 0.561 | 0.566 | 0.459 | 0.515 |
| P8 |  |  |  |  |  |  |  | 0.421 | 0.528 | 0.525 | 0.440 | 0.493 |
| P9 |  |  |  |  |  |  |  |  | 0.603 | 0.561 | 0.431 | 0.500 |
| P10 |  |  |  |  |  |  |  |  |  | 0.430 | 0.272 | 0.398 |
| P11 |  |  |  |  |  |  |  |  |  |  | 0.290 | 0.410 |
| P12 |  |  |  |  |  |  |  |  |  |  |  | 0.266 |

P1-P13: population ID

Table S2 Comparison of five demographic models for verifying inter-population hybridization between Pingshi and Danxiashan populations of *Viola hybanthoides*

| Model | MaxEstLhood | No. of params | AIC | ΔAIC |
| --- | --- | --- | --- | --- |
| M1 | -13490 | 5 | 26990 | 257 |
| M2 | -13446 | 5 | 26903 | 170 |
| M3 | -13359 | 7 | 26732 | 0 |
| M4 | -13392 | 7 | 26799 | 67 |
| M5 | -13456 | 6 | 26925 | 193 |

Notes: MaxEstLhood, maximum estimated log-likelihood; No. of params, number of estimated parameters; AIC, Akaike information criterion; ΔAIC, difference in AIC values relative to the optimal model with the lowest AIC value.
